# Clinical Characteristics and Mortality of Non-tuberculous Mycobacterial Infection in Immunocompromised vs. Immunocompetent Hosts

**DOI:** 10.3389/fmed.2022.884446

**Published:** 2022-05-18

**Authors:** Jingjing Chai, Xu Han, Qimin Mei, Tao Liu, Joseph Harold Walline, Jia Xu, Yecheng Liu, Huadong Zhu

**Affiliations:** ^1^Emergency Department, State Key Laboratory for Complex Severe and Rare Diseases, Peking Union Medical College Hospital, Peking Union Medical College, Chinese Academy of Medical Sciences, Beijing, China; ^2^Department of Pulmonary and Critical Care Medicine, Peking Union Medical College Hospital, Peking Union Medical College, Chinese Academy of Medical Sciences, Beijing, China; ^3^Accident and Emergency Medicine Academic Unit, Prince of Wales Hospital, The Chinese University of Hong Kong, Shatin, China; ^4^Department of Clinical Laboratory, Peking Union Medical College Hospital, Peking Union Medical College, Chinese Academy of Medical Sciences, Beijing, China

**Keywords:** non-tuberculous mycobacteria (NTM), mortality, comorbidity, immunocompromised, disseminated

## Abstract

Immunosuppression and host vulnerability play a key role in non-tuberculous mycobacteria (NTM) pathogenesis. The objective of this study was to compare the clinical characteristics and mortality of NTM infections in immunocompromised and immunocompetent patients. We used a retrospective dataset obtained from our large, tertiary, urban, teaching hospital which is the medical records of hospitalized patients with NTM infections between January 1, 2013 to December 31, 2020. The information including clinical manifestations, imaging, and NTM etiological data were obtained from the hospital's clinical data system. A total of 480 patients with NTM infections completed species identification. 118 hospitalized NTM patients who met ATS/IDSA NTM diagnostic criteria and had complete medical records were included in the study. The average age was 49.4 years, 57 (48.3%) were female, and 64 (54.2%) were immunosuppressed hosts. In our study, the most common species in order of frequency were: *M. intracellulare, M. abscessus, M. avium*, and *M. kansasii* among NTM patients. The most common comorbidity was history of previous tuberculosis (30.5%). Besides malignancy, the most common immunodeficiencies were adult-onset immunodeficiency induced by anti-interferon-gamma autoantibody, SLE, and vasculitis. The immunocompromised patients with NTM had more clinical symptoms, comorbidities and lower lymphocyte counts compared to immunocompetent patients. The mortality we observed in immunocompromised patients of NTM disease was significantly higher than that of immunocompetent patients (HR 3.537, 95% CI 1.526–8.362). Immunosuppressed NTM patients with lower B and CD4^+^ T lymphocyte counts may more frequently present with disseminated NTM infections, clinical exacerbations, and higher mortality than immunocompetent patients.

## Introduction

Non-tuberculous mycobacteria (NTM) include all species of mycobacteria except for M. tuberculosis and M. leprae and they are ubiquitous microorganisms that have an environmental origin. There are over 200 species of NTM identified to date, but only a few are pathogenic to susceptible hosts and are generally grouped with other opportunistic pathogens (1, 2). The incidence and prevalence of NTM disease are increasing in some countries and regions, even surpassing the incidence and prevalence of tuberculosis (3–6). The studies showed that the incidence of NTM lung disease increased in most U.S. states, Japan (6) and southern Taiwan (7). Winthrop K. L. showed that the annual incidence of NTM lung disease increased from 3.13 (95% CI, 2.88–3.40) to 4.73 (95% CI, 4.43–5.05) per 1,00,000 person-years in the United States (3). In Australia, the incidence of NTM infections has also increased (8).

Exposure to NTM species may cause pulmonary and extrapulmonary diseases. Chronic NTM pulmonary disease is the most common clinical manifestation. For a minority of susceptible persons, it may lead to extrapulmonary infections, including skin, joint, lymph node, and disseminated infections (9, 10). NTM infections can cause significant morbidity and mortality, however, and are often misdiagnosed as multiple drug-resistant mycobacterium tuberculosis (11, 12). Moreover, there are challenges with the treatment of NTM infections, such as a long course of treatment required to cure a patient, and how many patients experience a recurrence even after long treatment courses (13).

Susceptibility to infection is increased in an immunocompromised host because microorganisms such as NTM have weak natural virulence to person with normal immune defenses (14). The increased susceptibility to specific pathogens in various types of hosts, such as AIDS patients, solid organ transplant recipients, or patients receiving corticosteroids or other immunosuppressants, depends on the nature of their primary immune deficiency. Immunodeficiency is one of the important common predisposing factors in NTM disease (15). When assessing the risk of NTM infection, the patient's immune status should be thoroughly evaluated, if possible, including assessments of T lymphocytes, B lymphocytes, and inflammatory mediators, among others.

NTM infection has geographical distribution differences. Clinical manifestations and the prognosis of disease depend on the interaction of the host's immune response and the specific mycobacterial species. Host risk factors play an integral role in vulnerability to NTM disease (16). To the best of our knowledge, there is no relevant report in comparing characteristics and outcome between immunocompetent and immunocompromised patients in China. This study aimed to analyze the clinical symptoms and epidemiological characteristics of NTM infections in both immunocompromised and immunocompetent patients. By doing so, we hope to provide a reference for clinicians and microbiologists in diagnosing and predicting outcomes of NTM infection.

## Patients and Methods

### Study Population

We retrospectively reviewed the medical records of hospitalized patients who were diagnosed with “NTM disease” between January 1, 2013 and December 31, 2020 at a large, tertiary, urban, teaching hospital of Peking Union Medical College Hospital in Beijing, China. Data collected included patient characteristics, symptoms, medical histories, radiological findings, laboratory data since hospital admission, NTM species, clinical exacerbations, and comorbidities, which were obtained at the time of diagnosis and during follow-up.

### Inclusion Criteria and Definitions

Patients fulfilling criteria for one of the three major clinical syndromes of NTM infections based on a combination of clinical, radiographic, and microbiological criteria according to the guidelines of the American Thoracic Society and the Infectious Disease Society of America (ATS/IDSA), published in 2007 and updated in 2020 (9, 13), met inclusion for this study. At the same time, all the patients were treated for NTM disease with antibiotics according to guideline recommendation excluding colonization or contamination with NTM (13, 17).

In our study, the three major clinical syndromes caused by NTM infection depend on clinical presentation and the diagnostic criteria of the official ATS/IDSA statement:

1. Disseminated NTM disease
2. Pulmonary NTM Disease
3. Extrapulmonary NTM disease: lymphatic NTM disease, cutaneous NTM disease, skin, soft tissue, and bone NTM diseases.

Patients were divided into immunocompetent and immunocompromised categories according to their general state, medical history, medication, and immune status (18). Immunocompromised patients were defined broadly, including patients with acquired immunodeficiency syndrome (AIDS/HIV), those under continuous (>3 months) or high-dose (>0.5 mg/kg/day) corticosteroid or immunosuppressive therapy, solid-organ transplant recipients, those with rheumatic autoimmune diseases, solid-organ malignancies requiring chemotherapy in the last 5 years, those with any history of hematological malignancies, or those with primary immune deficiencies. Previous tuberculosis (TB) refers to patients who have previous treatment for active TB disease in the past history and were currently cured.

Charlson comorbidity index score was used to assess the comorbidities and known to be associated with mortality. The score includes myocardial infarction, diabetes mellitus, cancer, chronic pulmonary disease, liver disease, chronic renal failure, cerebrovascular accident, peptic ulcer, autoimmune disease, cirrhosis, leukemia, AIDS (19).

The date of NTM diagnosis was designated as the date of the first positive NTM culture. Findings and interval changes in clinical symptoms, bacteriological identification, and chest CTs during follow-up (from the initial screening or diagnostic images to the last observation) were assessed by physicians and radiologists together. The NTM exacerbations were judged based on the interval recurrence of symptoms and any changes in chest imaging during 1-year follow-up. We evaluated risk factors for all-cause mortality in NTM patients and calculated time-to-event as the time in days from the index of diagnosis until the date of death or end of follow-up (December 31, 2021), whichever occurred first for mortality analysis.

### Microbiologic Methods

Pathology detection methods included mycobacterium culture, acid-fast smear staining, immunological detection, DNA detection for NTM and mycobacterium tuberculosis (*MTB*). Samples sent for examination included sputum, peripheral blood, tracheobronchial aspirates, bronchoalveolar lavage fluid, stool, pus, wound secretions, pleural effusions, ascites fluid, subcutaneous nodules, skin tissue, joint fluid, lymph node biopsies, subcutaneous tissue or bone marrow.

Diagnostic steps were as follows: (1) mycobacterium culture: the fully automatic BACTEC MGIT 960 Mycobacterium culture system (Becton, Dickinson U.K.Limited, Berkshire, UK, BD) was used to identify mycobacteria. Cultures can be positive after 2–4 weeks of culture but were reported negative after 42 days of no growth. Samples of culture-positive mycobacteria were screened, smeared and inoculated with Mycobacterium Roche Medium for preliminary mycobacterium identification (20). It is used to distinguish patients with and without mycobacterial infection (2) immunological detection method: after mycobacterium detection on culture, the immunological monitoring method and the MPB64 antigen colloidal gold method were used to monitor for antigens of *MTB*. If this antigen monitoring was negative, the sample was considered to be NTM. After the initial differentiation, molecular testing was performed to determine the type of mycobacteria (3). Molecular detection: NTM or *MTB* were then confirmed by polymerase chain reaction (PCR) and NTM species identification was carried out by chip hybridization or DNA sequencing. NTM species identification was performed using gene chip (CapitalBio Corporation, Chengdu, China). The DNA microarray chip method was used to identify the common clinical mycobacteria species. We performed *rpoB* DNA sequencing to identify the rare NTM species. The DNA sequencing service was provided by gene sequencing company (RuiBiotech Corporation, Beijing, China).

### Ethics Statement

This study conformed to the Declaration of Helsinki and was approved by the medical ethics committee of the host institution (ethics approval number: S-K1673).

### Statistical Analysis

Statistical analysis was conducted using SPSS 22.0 software (SPSS Inc., Chicago, IL, USA) and GraphPad Prism 6.0 (GraphPad Software, La Jolla, CA). The Kolmogorov-Smimov method was used to test whether the data conformed to a normal distribution. Continuous variables with normal distribution were expressed as means ± standard deviations (SD), while continuous variables with non-normal distribution were expressed as medians with interquartile ranges. Categorical variables were expressed using frequencies and percentages. A non-parametric test (the Mann–Whitney *U* test or Kruskal–Wallis test) was used for analyzing data with non-normal distributions or heterogeneity of variances. Categorical variables were used with the Chi-square test or Fisher's exact test.

We analyzed demographic, clinical, and laboratory data to identify predictors of all-cause mortality among patients with NTM. Specifically, we considered age, gender, body mass index (BMI), comorbidities, and lymphocyte count, immunosuppression, disseminated NTM infection, and hemoglobin (Hgb) levels. The univariate and multivariate association of demographic, clinical, and laboratory test factors with risk of mortality was assessed using Cox proportional hazards regression models. Hazard ratios (HRs) were calculated using the Cox proportional hazard model. Statistical significance was assessed at *P* < 0.05. Kaplan-Meier survival curves were calculated, with the significance of difference in the survival distribution function estimated using the log rank test.

## Results

### Flow Chart and NTM Species Identification

**Figure 1:**
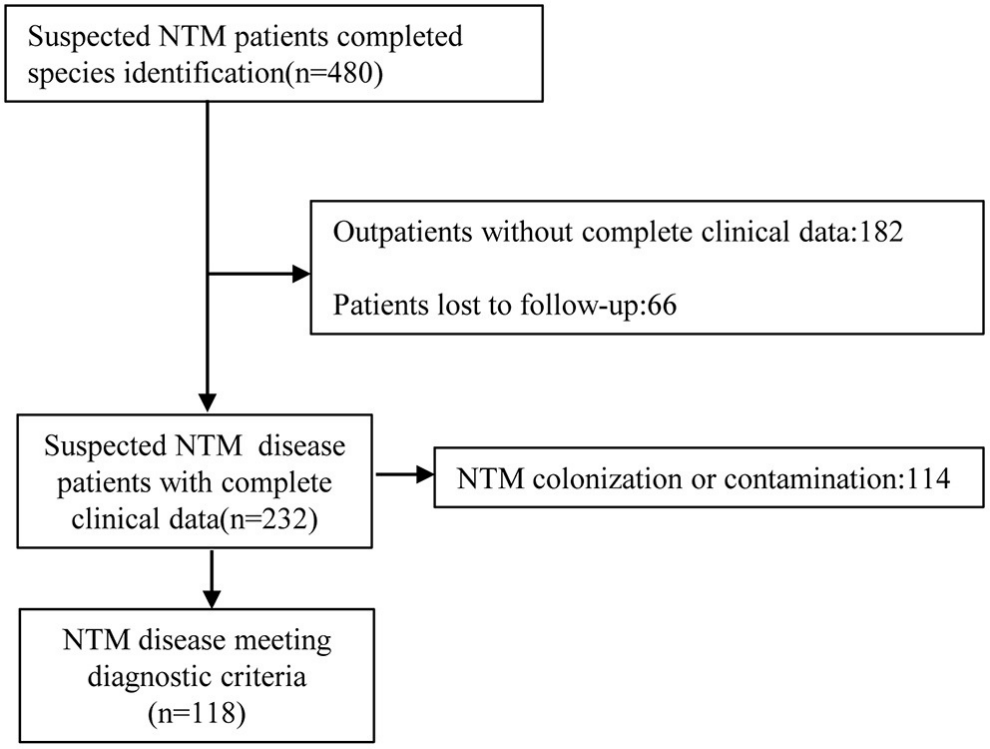
Study flow chart.

A total of 480 suspected NTM patients completed species identification between January 1, 2013 and December 31, 2020 and we found a total of 38 NTM species. After reviewing microbiological results, clinical characteristics, and radiologic findings, there were 118 NTM patients who met ATS/IDSA NTM disease diagnostic criteria and had complete medical records were included for further analysis (Figure 1).

**Figure 2:**
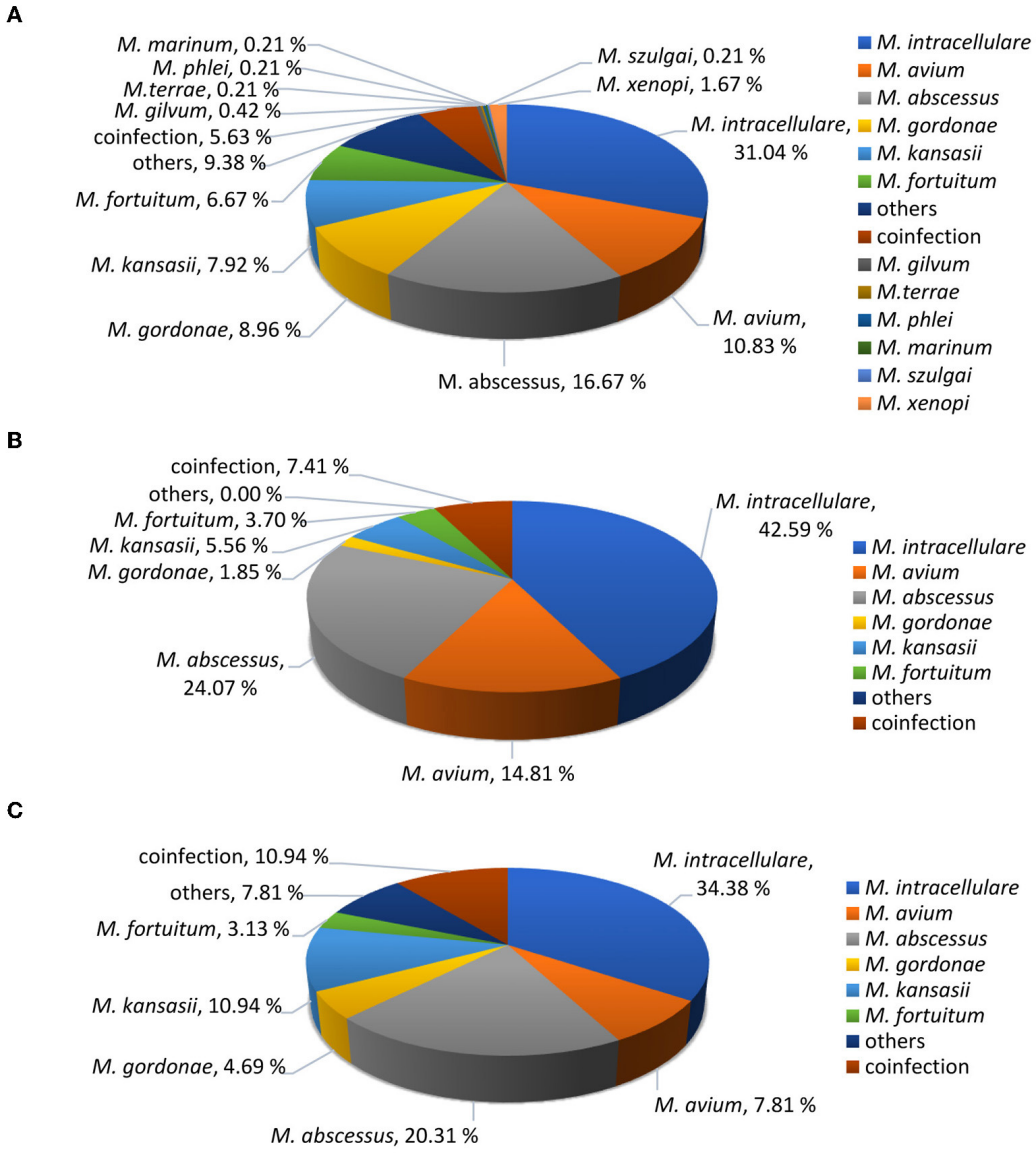
Identified NTM species percentages. **(A)** Total suspected NTM patients (*n* = 480). **(B)** Diagnosed NTM infections in immunocompetent patients (*n* = 54). **(C)** Diagnosed NTM infections in immunocompromised patients (*n* = 64).

Among the 118 patients, 11 NTM species were identified. The most common species were *M. intracellulare* (45/118, 38.1%), followed by *M. abscessus* (26/118, 22.0%), *M. avium* (13/118, 11.0%) and *M. kansasii* (10/118, 8.5%). Others included *M. gordonae* (4/118, 3.4%), *M. fortuitum* (4/118, 3.4%) *and coinfection* (11/118, 9.32%). The rare NTM included *M. xenopi* (1/118, 0.8%), *M. szulgai* (1/118, 0.8%), *M. colombiense* (1/118, 0.8%), *M. paraseoulense* (1/118, 0.8%) and *M. lentiflavum* (1/118, 0.8%). These rare NTM species were all found in immunocompromised patients. We compared the percentage of species between immunosuppressed and immunocompetent hosts. There was no significant difference in NTM species identification between immunosuppressed and immunocompetent patients (*P* = 0.224), and the percentage of mycobacteria species were shown in Figure 2.

### Patients' Immune Status

Patients were classified according to their immune status, as shown in Table 1. Among them, primary immunodeficiency diseases were found in 10 patients: three patients with cellular immune dysfunction, five patients with adult-onset immunodeficiency, one patient with MonoMAC syndrome, and one patient with hypoimmunoglobulinemia.

**Table 1:** Characteristics and predisposing conditions in patients with NTM infections (*n* = 118).

**Underlying immune disease**	***n***	**Percentage**
Immunocompetent	54	45.8%
Immunocompromised	64	54.2%
AIDS	4	3.4%
**Rheumatic autoimmune diseases**		
SLE	5	4.3%
RA	4	3.4%
Vasculitis	5	4.3%
PM/DM	1	0.8%
SSc	2	1.7%
Overlap syndrome	1	0.8%
pSS	2	1.7%
Ankylosing spondylitis	1	0.8%
Relapsing polychondritis	1	0.8%
Anti-synthetase antibody syndrome	1	0.8%
**Malignancy**		
Solid-organ malignancy in chemotherapy	4	3.4%
Haematologic malignancy	5	4.3%
Others under continuous corticosteroid (>3 months or high-dose > 0.5 mg/kg/day) or immunosuppressive therapy	18	15.3%
**Primary immunodeficiency diseases**		
Cellular immune dysfunction	3	2.5%
Adult-onset immunodeficiency	5	4.3%
MonoMAC syndrome	1	0.8%
Hypoimmunoglobulinemia	1	0.8%

*AIDS, acquired immunodeficiency syndrome; SLE, systemic lupus erythematosus; RA, rheumatoid arthritis; PM, polymyositis; DM, dermatomyositis; SSc, systemic sclerosis; Overlap syndrome, includes features of SSc and PM; pSS, primary Sjögren syndrome*.

### Patient Characteristics and Clinical Manifestations

A comparison of patient characteristics and clinical manifestations between immunocompetent and immunocompromised patients with NTM infections is shown in Table 2. NTM patients in immunocompromised group had more comorbidities compared to immunocompetent group: chronic kidney disease (17.2 vs. 3.7%) (*P* = 0.02), fever (87.5 vs. 66.7%) (*P* = 0.007), bone and joint pain (25 vs. 5.6%) (*P* = 0.004), disseminated NTM infection (35.9 vs. 11.1%) (*P* = 0.002). Immunocompromised patients also had more frequent exacerbations than immunocompetent patients (59.4 vs. 27.8%) (*P* = 0.001).

**Table 2:** Comparison of characteristics and clinical manifestations between immunocompetent and immunocompromised patients with NTM infections.

**Variables**	**Total (*n =* 118)**	**Immune status**		***P-*** **value**
		**Immunocompetent (*n =* 54)**	**Immunocompromised (*n =* 64)**	
Age, mean ± SD	49.4 ± 15.6	45.8 ± 15.2	52.6 ± 15.2	0.017
Gender, *n* (%)				
Female	57 (48.3)	27 (50.0)	30 (46.9)	0.735
Male	61 (51.7)	27 (50.0)	34 (53.1)	0.735
BMI, mean ± SD (kg/m^2^)	20.9 ± 3.4	21.5 ± 3.6	20.4 ± 3.1	0.057
Time from onset to diagnosis (m), median (IQR)	13.0 (4.7–35)	12.1 (4.1–42.8)	14.5 (5.0–32.0)	0.785
Smoking, *n* (%)				
Smoker	45 (38.1)	21 (38.9)	24 (37.5)	0.877
Ex-smoker	31 (26.3)	15 (27.8)	16 (25.0)	0.103
Comorbidity, *n* (%)				
previous tuberculosis	36 (30.5)	17 (31.4)	19 (29.7)	0.973
Bronchiectasis	25 (21.2)	16 (29.6)	9 (14.1)	0.137
COPD	4 (3.4)	1 (1.8)	3 (4.7)	0.394
DPLD	14 (11.9)	4 (7.4)	10 (15.6)	0.132
Diabetes mellitus	13 (11.0)	3 (5.6)	10 (15.6)	0.082
Cerebrovascular disease	2 (1.7)	1 (1.9)	1 (1.6)	0.903
Chronic kidney disease	13 (11.0)	2 (3.7)	11 (17.2)	0.020^**^
Chronic liver disease	11 (9.3)	4 (7.4)	7 (10.9)	0.511
AIDS	4 (3.4)	0 (0)	4 (6.3)	0.053
Medication, *n* (%)	35 (29.7)	0 (0)	35 (54.7)	0.000^**^
Clinical symptoms, *n* (%)				
cough	86 (72.9)	38 (70.4)	48 (75)	0.573
Sputum	76 (64.4)	33 (61.1)	43 (67.2)	0.492
hemoptysis	22 (18.6)	13 (24.1)	9 (14.1)	0.164
chest pain	19 (16.1)	8 (14.8)	11 (17.2)	0.727
chest tightness	41 (34.7)	16 (29.6)	25 (39.1)	0.284
dyspnea	56 (47.5)	23 (42.6)	33 (51.6)	0.331
fever	92 (78.0)	36 (66.7)	56 (87.5)	0.007^**^
fatigue	34 (28.8)	14 (25.9)	20 (31.2)	0.737
weight loss	58 (49.2)	29 (53.7)	29 (45.3)	0.364
night sweats	14 (11.9)	6 (11.1)	8 (12.5)	0.816
bone and joint pain	19 (16.1)	3 (5.6)	16 (25.0)	0.004^**^
enlarged lymph nodes	32 (27.1)	12 (22.2)	20 (31.3)	0.249
Pulmonary NTM	105 (89.0)	50 (92.6)	55 (85.9)	0.250
Extrapulmonary NTM	13 (11.0)	4 (7.4)	9 (14.1)	0.250
Disseminated NTM infection	29 (24.6)	6 (11.1)	23 (35.9)	0.002^**^
WBC^$^, median (IQR) × 10^6^/L	7.1 (4.7–11.5)	6.8 (4.5–11.2)	7.2 (4.9–12.2)	0.303
Lymphocyte count^$^, median (IQR) × 10^6^/L	1.3 (0.7–1.8)	1.5 (1.1–1.9)	1.1 (0.6–1.4)	0.001^&^
Hgb^$^, median (IQR) g/L	109.0 (88.0–129.2)	120.5 (94.7–136.5)	101.5 (86.2–115.7)	0.002^&^
Lymphocyte subsets^*^ (*n* = 76) (/μL)				
B lymphocyte	60.5 (18.2–138.2)	97.2 (35.0–196.5)	43.0 (15.2–83.7)	0.006^&^
T lymphocyte	829.0 (420.5–1145.0)	938.0 (435.5–1630.0)	746.0 (376.5–1027.0)	0.026^&^
CD4^+^ T lymphocyte	419.0 (156.7–575.2)	545.5 (336.0–1023.0)	310.5 (139.7–484.2)	0.001^&^
CD8^+^ T lymphocyte	318.0 (197.5–539.7)	321.5 (213.2–554.7)	309.5 (162.2–524.0)	0.238
NK lymphocyte	124.0 (38.0–233.0)	120.0 (52.5–232.5)	126.5 (29.7–236.0)	0.585
CD4^+^ /CD8^+^ T lymphocyte ratio	1.3 (0.7–1.7)	1.5 (0.8–2.0)	1.2 (0.6–1.6)	0.023^&^
Serum IgG^$^, median (IQR) × 10^3^mg/dL	12.1 (8.8–16.4) (*n* = 106)	11.8 (9.6–14.4) (*n* = 47)	12.7 (8.7–19.8) (*n* = 59)	0.407
Exacerbation	53 (44.9)	15 (27.8)	38 (59.4)	0.001^**^

*NTM, non-tuberculous mycobacteria; IQR, interquartile range; BMI, body mass index; SD, standard deviation; COPD, chronic obstructive pulmonary disease; DPLD, diffuse parenchymal lung disease; IgG, immunoglobulin G; WBC, white blood cells; Hgb, hemoglobin; m, months; medication, glucocorticoids or immunosuppressive agents;*

* *Lymphocyte subsets (n = 76), immunocompetent group (n = 30), and immunocompromised group (n = 46);*

$ *, at NTM diagnosis;*

** *Chi-square test (P < 0.05).*

& *Mann-Whitney test (P < 0.05)*.

### Charlson Comorbidity Index Score Comparison

**Figure 3:**
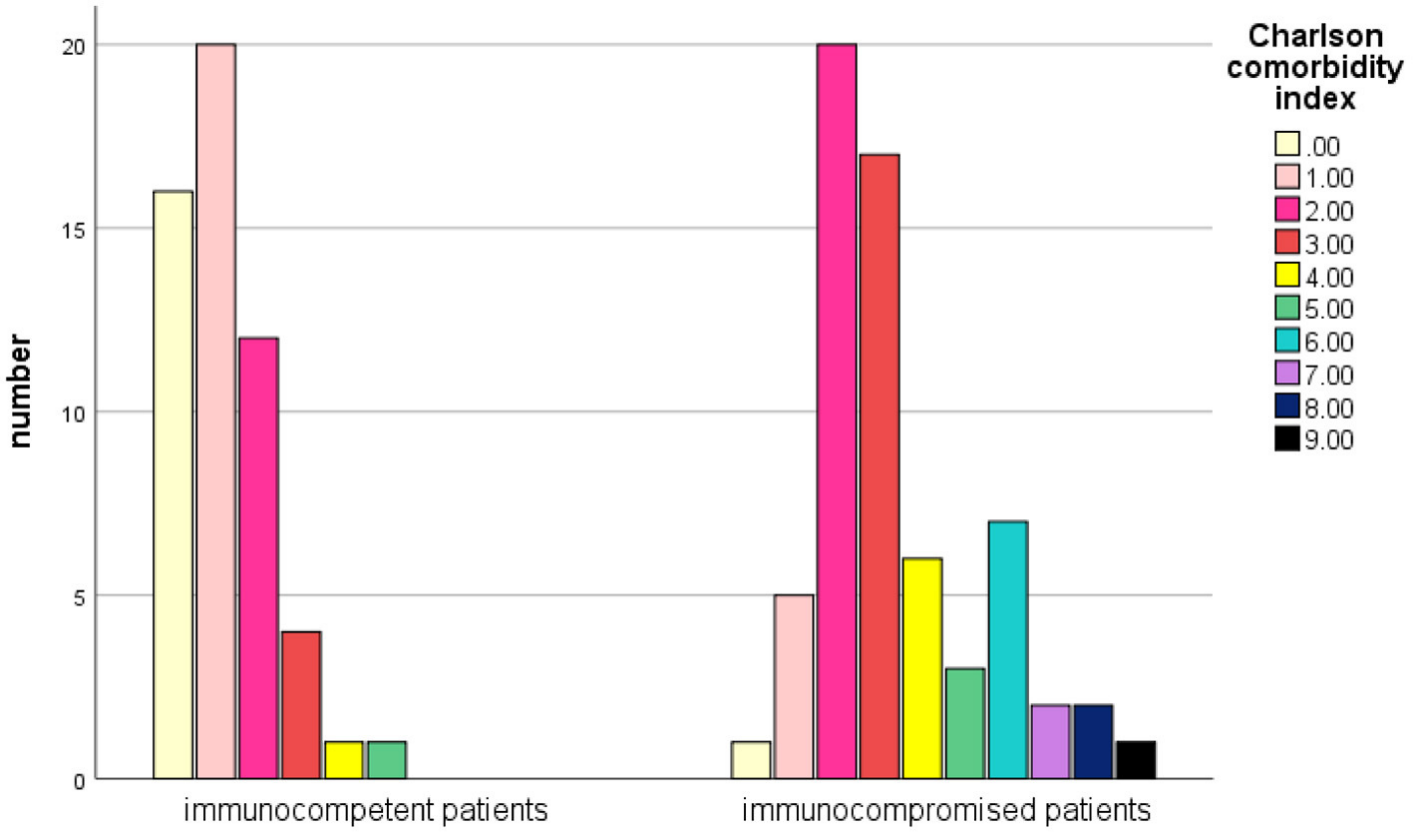
Comparison of the Charlson Comorbidity Index score between immunocompetent and immunosuppressive patients with NTM infections.

The mean Charlson Comorbidity Index (CCI) score (19) was 2.38 in total patients, and 1.2 in immunocompetent patients (vs. 3.4 in immunocompromised patients, *P* < 0.001) (Figure 3). 37.3% (44/118) of those patients had a CCI score of 3 or higher.

### Mortality and Survival Comparison

In 118 NTM patient, 30 (25.4%) patients died during the follow-up period. Among them, 8 (14.8%) cases and 22 (34.3%) cases were from immunocompetent and immunocompromised patients, respectively. The median age at death was 50 years. The median length of follow-up was 41 month. The overall mortality rate was 9.6 per 100 person-years. Cumulative mortality was 16.9% at 1 years, 24.5% at 3 years and 25.4% at 5 years in total patients, 7.4% at 1 years, 12.9% at 3 years and 12.9% at 5 years in immunocompetent patients, and 25% at 1 years, 34.3% at 3 years and 35.9% at 5 years in immunocompromised patients, respectively. The all-cause mortality rate is higher and earlier in immunocompromised patients than immunocompetent patients.

Of the 30 patients, 23 (76.7%) patients died at the end of the observation whose primary cause of death was thought to be NTM disease or related to NTM infection. Among them, nine patients had respiratory failure due to pulmonary multiple infections including NTM: five cases with aspergillus, one case with actinomycete, one case with staphylococcus aureus, one case with Burkholderia cepacia, one case with pseudomonas aeruginosa. Other causes of death included one patient died of acute myocardial infarction, one died of gastrointestinal bleeding, three died of malignant tumor, two died of pulmonary alveolar proteinosis.

**Figure 4:**
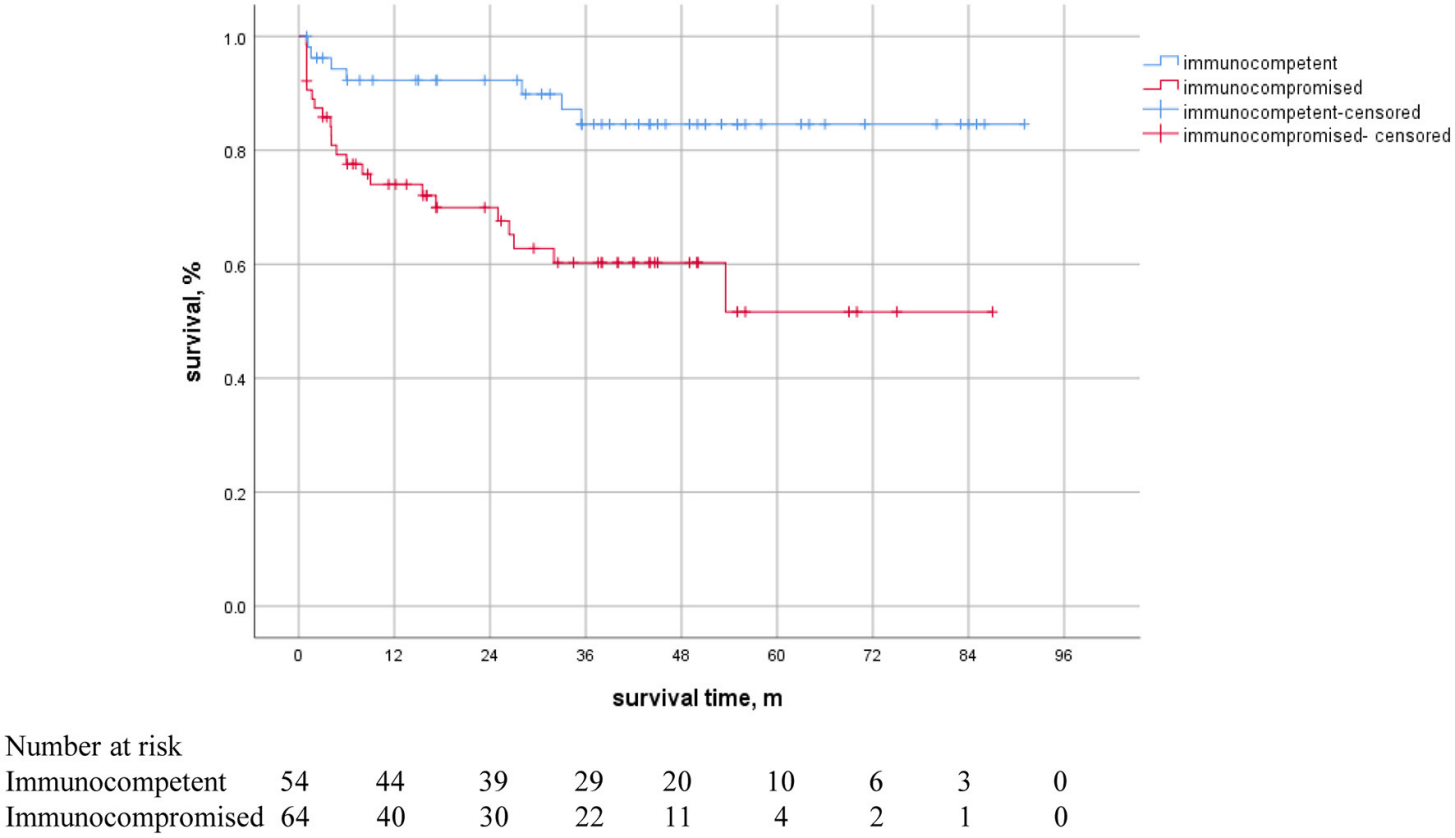
Kaplan-Meier survival curves in NTM patients with or without immunosuppression. There was a statistically significant difference between the curves (HR 3.537, 95% CI 1.526–8.362, *P* = 0.003).

A Kaplan-Meier survival curve analysis showed that all-cause mortality in immunocompromised patients was significantly higher than that in immunocompetent patients (Figure 4). There is a statistically significant difference between curves (reference category = immunocompetent, HR 3.537, 95%CI 1.526–8.362, *P* = 0.003, *log-rank*) in crude survival comparison.

### Risk Factors for Death

**Figure 5:**
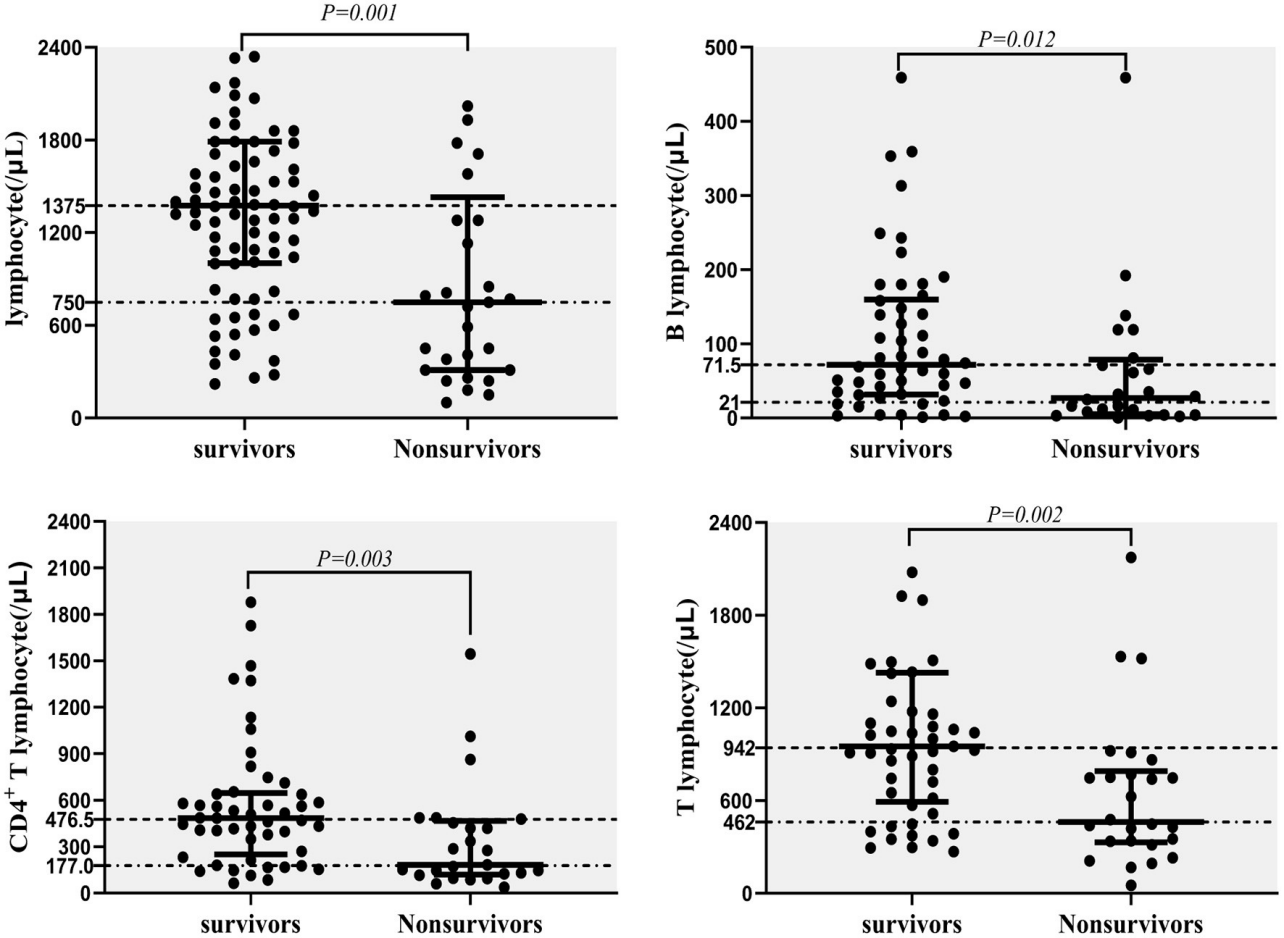
Predictors for mortality in NTM disease; lymphocyte counts (/μL), T lymphocyte counts (/μL), CD4^+^ T lymphocyte counts (/μL), and B lymphocyte counts (/μL) in survivors and non-survivors.

The CD4^+^ T lymphocyte counts, as well as lymphocyte, T lymphocyte and B lymphocyte counts (/μL), were significantly lower in non-survivors compared with survivors (Figure 5). These data were measured at diagnosis of NTM disease.

Cox proportional hazard regression analyses was conducted to assess risk factors related to all-cause mortality, including baseline factors (age, gender, BMI, and CCI), immunosuppression, disseminated NTM infection, CD4^+^ T lymphocyte count, B lymphocyte count, and Hgb at the time of NTM diagnosis. The univariate model included each factor separately, and the multivariate models included these potential predictors simultaneously. CD4^+^ T lymphocyte count were correlated with B lymphocyte count (Pearson correlation coefficient = 0.364, *P* = 0.001) and immunosuppression, so they were analyzed separately. In univariable analysis, the prognostic factors that appeared to be significantly associated with mortality included immunosuppression, disseminated NTM infection, CD4^+^ T lymphocyte count, B lymphocyte count, and Hgb. In multivariate analysis, after adjust age, gender, BMI, CCI, disseminated NTM infection, the result showed that decreased CD4^+^ T and B lymphocyte count and decreased Hgb were all independent predictors of mortality in NTM patients (Table 3).

**Table 3:** Factors associated with all-cause mortality in NTM patients.

**Variables**	**Univariate**			**Multivariate**		
	**HR**	**95% CI**	***P-*** **value**	**HR**	**95% CI**	***P-*** **value**
B lymphocyte (/μL)						
<18	7.130	1.571–32.364	0.011	6.354	1.339–30.149	0.020
18–59	2.324	0.424–12.745	0.331	1.792	0.311–10.332	0.541
60–138	3.624	0.752–17.458	0.109	2.619	0.523–13.118	0.242
>138	1 [Reference]			1 [Reference]		
CD4^+^ T lymphocyte (/μL)						
<156	5.546	1.554–19.795	0.008	4.974	1.293–19.130	0.020
156–419	3.264	0.837–12.731	0.088	2.926	0.738–11.608	0.127
420–575	1.709	0.382–7.650	0.484	1.634	0.354–7.542	0.529
>575	1 [Reference]	-	-	1 [Reference]		
Hgb (<109 g/L)	5.769	2.205–15.093	0.000	3.262	1.027–10.366	0.045
Dissemination NTM infection	2.518	1.219–5.202	0.013	NA		
Immunosuppression	3.537	1.526–8.362	0.003	NA		

*NTM, non-tuberculous mycobacteria; Hgb, Hemoglobin; BMI, body mass index (calculated as weight in kilograms divided by height in meters squared); HR, hazard ratio; CI, confidence interval; CCI, Charlson Comorbidity Index*.

## Discussion

This retrospective cohort study described the characteristics, etiology, comorbidities, and mortality of patients with NTM infections with and without immunocompromise in a real-world setting in China. In our study, the results show that NTM disease with immunosuppression have a higher CCI score, more disseminated NTM infections, higher rates of clinical exacerbations, and higher mortality rates with lower B and T lymphocyte counts than immunocompetent patients. It has long been known that infection risk is closely related to a patient's immune status, and immunocompromised individuals are vulnerable to many opportunistic pathogens such as NTM (21). Immunocompromise and host vulnerability appear to play a central role in NTM pathogenesis and increased risk for poor outcomes (22).

NTM is ubiquitous in the environment and grows widely in water, soil, and animal sources. NTM infections did not receive much attention in the past and were dismissed as laboratory contamination (13, 20). With the development of more advanced laboratory techniques and the strengthening of clinicians' awareness, the global reported incidence of NTM disease has been increasing over the past decade (8, 10). Other causes may include the aging of the population and the increased number of immunosuppressed people (23). As a receiving center for many challenging infectious and autoimmune diseases, our hospital has a uniquely large dataset of NTM infections. To our knowledge, this is the first study specifically exploring differences in NTM infections between immunosuppressed and immunocompetent patients in China. We investigated the clinical characteristics and outcomes over the past 8 years, and explored prognostic factors of mortality in NTM patients. The results of this study showed that more than half of NTM patients were immunosuppressed. This fraction of immunocompromised patients is higher than that reported in other literature (24). It may be because our institution has a large number of patients with immune-related diseases compared to other hospitals. Nearly one-fifth of patients were suffering from rheumatic autoimmune diseases. Besides malignancy, the most common immunodeficiency disease was adult-onset immunodeficiency (25), SLE and vasculitis. It has been reported that NTM pulmonary diseases was associated with rheumatic autoimmune diseases such as rheumatoid arthritis and Sjögren syndrome (21, 26, 27). These discrepancies may be due to epidemiologic differences among studies.

The distribution of NTM pathogens is geographically specific. In our research, *M. intracellulare* was the predominant NTM species identified, consistent with the results of Wang C.F in Changchun district (28) and Hu C.M. in Nanjing district (29). However, the predominant NTM species were identified to be *M. abscessus* complex in Chongqing district (30). Pang Y. previously reported that *M. abscessus* was the predominant species in eastern and southern China (31). These results may be related to climate variability between northern and southern China. In Japan, the most common species among NTM pulmonary disease was *Mycobacterium avium complex* (87.3%), followed by *M. abscessus complex* (5.5%), *M. kansasii* (3.9%), and *M. fortuitum* (1.3%) (6). In America, *M. avium* was more frequently isolated (68.5%) than *M. intracellulare* (6.6%). In Africa and Oceania, *M. intracellulare* was more common (77% and 79.5%, respectively). *M. abscessus* complex was frequently isolated in Asia (16%) and Oceania (12%), in contrast to Europe (2.9%), North America (3.2%) and South America (5.7%) (32). Notably, we observed that rare NTM species were all detected in immunocompromised patients. In our study among NTM patients, the most common comorbidity was a history of tuberculosis, which was significantly higher than that in Western countries and similar to previously published results from Taiwan (7).

As shown in this study, compared with immunocompetent patients, immunosuppressed patients often presented with more clinical symptoms of fever, and bone and joint pain, which are two types of characteristics of rheumatic autoimmune diseases. The reasons for this may be two-fold: NTM infections may induce immune disease activity and NTM infections in immunosuppressed patients are not easy localizable, leading to more disseminated disease with more severe infections. In laboratory tests, immunosuppressed patients had lower B and T lymphocyte counts, especially CD4^+^ T lymphocyte counts, compared with immunocompetent patients. Lapinel N. C. showed that HIV patients might be vulnerable to the consequences of NTM because of their defective T cell-mediated immunity (33). Peripheral blood lymphocyte subsets especially CD4^+^ T and B lymphocytes may be used as one of the main indicators for judging the initial immune status and outcome for NTM patients. Mourad A.'s research showed that comorbidities did not explain all of the reduction in expected survival among 653 patients with NTM-pulmonary disease (24). According to our cox regression analysis results, decreased CD4^+^ T and B lymphocyte count and decreased Hgb were independent predictors of all-cause mortality in NTM patients after adjust CCI and other factors. The results suggest that in addition to comorbidities, physicians should pay more attention to the patient's immune status, especially CD4^+^ T and B lymphocyte counts. These factors may be associated with high risk of death in NTM patients. The mortality we observed in immunocompromised patients was significantly higher than that in immunocompetent patients. The overall mortality rate is higher than the result of Fleshner's study (34). The reason for consideration may be that many patients have severe immunodeficiency. Disseminated NTM infections, CD4^+^ T and B lymphocyte count, and lower Hgb levels are all associated with a higher risk for death. These factors may be used to assist in predicting the mortality of patients with NTM infections. Interestingly, we also found that low Hgb levels were associated with a high risk of death in NTM patients. Ye SS previously showed that laboratory tests of anemia in NTM patients were associated with disseminated NTM disease (35). Additional research found that HIV patients with decreased CD4^+^ T lymphocyte counts (<50/μL) were more likely to progress to disseminated NTM disease (36, 37). NTM patients with lower lymphocyte counts may have a variety of immune dysfunctions including innate immunity, humoral immunity, or cellular immunity that may lead to poorer prognosis and higher mortality.

### Limitations

Several limitations in this study should be acknowledged. This was a single-center retrospective study, which has its inherent limitations. The sample size included in our study was relatively small, and the result may only be representative of a subset of the population. Additionally, only about a quarter of NTM patients were able to complete analysis due to a lack of complete medical records. This was a conscious choice on our part, as we wanted to maximize the number of patients with complete data, rather than the total number of patients alone. NTM infections are quite rare, so being able to find over 100 cases with full data for analysis is still remarkable and has certain representativeness. Finally, we didn't include factors such as drug combinations, duration of medications, or patient compliance during NTM infection, which may affect patient outcomes. Future work should include prospective, controlled studies to evaluate prognosis and confirm our findings.

## Conclusion

The patient's immune status could affect the occurrence and evolution of NTM disease. Compared to immunocompetent patients with NTM infection, immunosuppressed patients with lower B and CD4^+^ T lymphocyte counts may more frequently present with disseminated NTM infections, and have poorer prognoses with higher mortality rates.

## Data Availability Statement

The raw data supporting the conclusions of this article will be made available by the authors, without undue reservation.

## Ethics Statement

The studies involving human participants were reviewed and approved by the Medical Ethics Committee of Peking Union Medical College Hospital. Written informed consent for participation was not required for this study in accordance with the national legislation and the institutional requirements.

## Author Contributions

HZ and YL conceived the idea. JC, XH, and QM collected the data. JC, YL, and TL performed the analysis and interpreted the results. JC, YL, and JW drafted the manuscript and made revisions. YL and HZ supervised the study and made the decision for submission. All the authors reviewed and approved the final manuscript.

## Funding

This study was supported by the National Key Research and Development Program of China (2021YFC2501800).

## Conflict of Interest

The authors declare that the research was conducted in the absence of any commercial or financial relationships that could be construed as a potential conflict of interest.

## Publisher's Note

All claims expressed in this article are solely those of the authors and do not necessarily represent those of their affiliated organizations, or those of the publisher, the editors and the reviewers. Any product that may be evaluated in this article, or claim that may be made by its manufacturer, is not guaranteed or endorsed by the publisher.

## Abbreviations

NTM: non-tuberculous mycobacteria
MTB: mycobacterium tuberculosis
TB: tuberculosis
ATS/IDSA: American Thoracic Society and the Infectious Disease Society of America
HR: hazard ratio
CCI: Charlson Comorbidity Index
AIDS: acquired immunodeficiency syndrome
HIV: human immunodeficiency virus
Hgb: hemoglobin
SLE: systemic lupus erythematosus.
